# Identification of ecological networks and nodes in Fujian province based on green and blue corridors

**DOI:** 10.1038/s41598-021-99416-4

**Published:** 2021-10-22

**Authors:** Qingqing Zhou, Cecil C. Konijnendijk van den Bosch, Jingru Chen, Wenbing Zhang, Jianwen Dong

**Affiliations:** 1 College of Landscape Architecture, Fujian Agriculture and Forestry University, Fuzhou, 350002 China; 2 China Urban Construction Design and Research Institute Co., Ltd, Beijing, 100011 China; 3 The Lab of Urban Forestry Research in Action, Department of Forest Resources Management, The University of British Columbia, 2329 West Mall, Vancouver, V6T 1Z4 Canada; 4 Department of Arts and Design, Xiamen University Tan Kah Kee College, Zhangzhou, 363105 China

**Keywords:** Ecology, Environmental sciences

## Abstract

China's Green Space System Planning (GSSP) research has gradually expanded from central urban areas to municipal and provincial scales in recent years. Besides, the research on the role of green space in the water environment has also attracted much attention. However, the study of green corridors usually ignored hydrological data, which widespread absence especially in the large area scale. And the scale of green corridor construction mainly focused on central urban areas. This paper took China's Fujian province as an example. Based on the DEM elevation data, the article identified blue corridors without hydrological data. In addition, the green corridors were determined based on the land use data. According to the green corridors and blue corridors protection, we identified the ecological networks and nodes by the network analysis method. The results showed that the blue corridors identified by DEM data were consistent with the hydrological status quo. The regional status of the identified ecological networks and nodes were basically in line with their characteristics, proving the value of the planning methods. Finally, based on the identification results, suggestions for Fujian's ecological networks and nodes are put forward.

## Introduction

As an essential part of the terrestrial ecosystem, green space plays an important role in promoting human health, urban construction, and in regional ecology^1–3^. However, related studies have shown that human activities in the process of urbanization have had a substantial negative impact on the green space ecosystem, especially the acceleration in the fragmentation of the ecological landscape^4–6^.

In China, the Green Space System Planning (GSSP) has become an important type of Special Plan, providing an essential basis for ecological protection and green space construction^7,8^. However, green spaces in urban areas have always been key research topics. In recent years, China has promoted the construction of a so-called ecological civilization, and the focus of GSSP has expanded from urban areas to city areas. Some cities have already made some explorations in the identification of city ecological corridors. Research on local green infrastructure, rainwater systems, and city green corridors has generated important findings^9–13^. However, in many countries there is less experience with green infrastructure planning at the provincial level. Two knowledge gaps in particular can be highlighted.

First, there are few studies at the provincial scale in countries like China. The provincial level has always been a ‘blind spot’ in China's GSSP. In 2019, the Chinese government issued a policy document^14^ requiring all parts of the country to compile Territorial Space Planning (TSP). As an essential part of TSP, GSSP is divided into city-level GSSP and urban-level GSSP^8,15,16^. In 2020, China has required the provinces to formulate provincial TSP^17^, and the provincial level of GSSP has also become a new research area of GSSP. At the same time, the research of provincial ecological corridors and nodes has become an essential part of the provincial GSSP.

In 2020, Guidelines for Formulation of Provincial Territorial Space Planning ^17^ were issued, and these have become the only official document that guides Provincial TSP so far. Large-scale ecological protection instead of urban construction has become the dominant factor, and the provincial GSSP as a significant component of provincial TSP has received more attention. The scientific identity and construction of ecological networks and ecological nodes have become an essential work of the provincial GSSP.

A second knowledge gap relates to only few studies on GSSP’s ecological networks having focused on protecting green–blue corridors. At present, the existing research on GSSP ecological corridor mainly focuses on biological migration^18^, resilience^19^, vulnerability index^20^, etc. In addition, many studies have pointed out that protecting the natural water system has a significant and positive effect on the regional ecological environment^21,22^. However, due to the general lack of primary hydrological data, there are rarely researches on the blue network at the provincial level in China. Many researches are focus on integrated rainwater management^23,24^ and the impact of water bodies on the microclimate^15,16^. As China’ s planning concept of Sponge Cities has been upgraded from a pilot concept to an essential requirement for general city construction, protecting the green–blue corridors has become one of the primary tasks of the GSSP. The blue network in this paper is composed of mainstream, tributary, and surface runoff corridors. We use Digital Elevation Models (DEM), the open-source elevation data, to identify blue corridors to deal with the lack of hydrological data. Analyzing land use data and DEM with GIS, we looked at Fujian Province as a case study for identifying green and blue corridors.

Based on the green–blue corridors, ecological network and nodes are identified. Next, based on the current green–blue corridors, suggestions are made for the development of the ecological network. More specifically, this paper addresses the following questions: (1) How can green and blue corridors be identified at the provincial level? and (2) how can a sound basis for provincial ecological networks and nodes be based on proper identification of green and blue corridors?

## Study area, materials, and methods

### Study area

**Figure 1:**
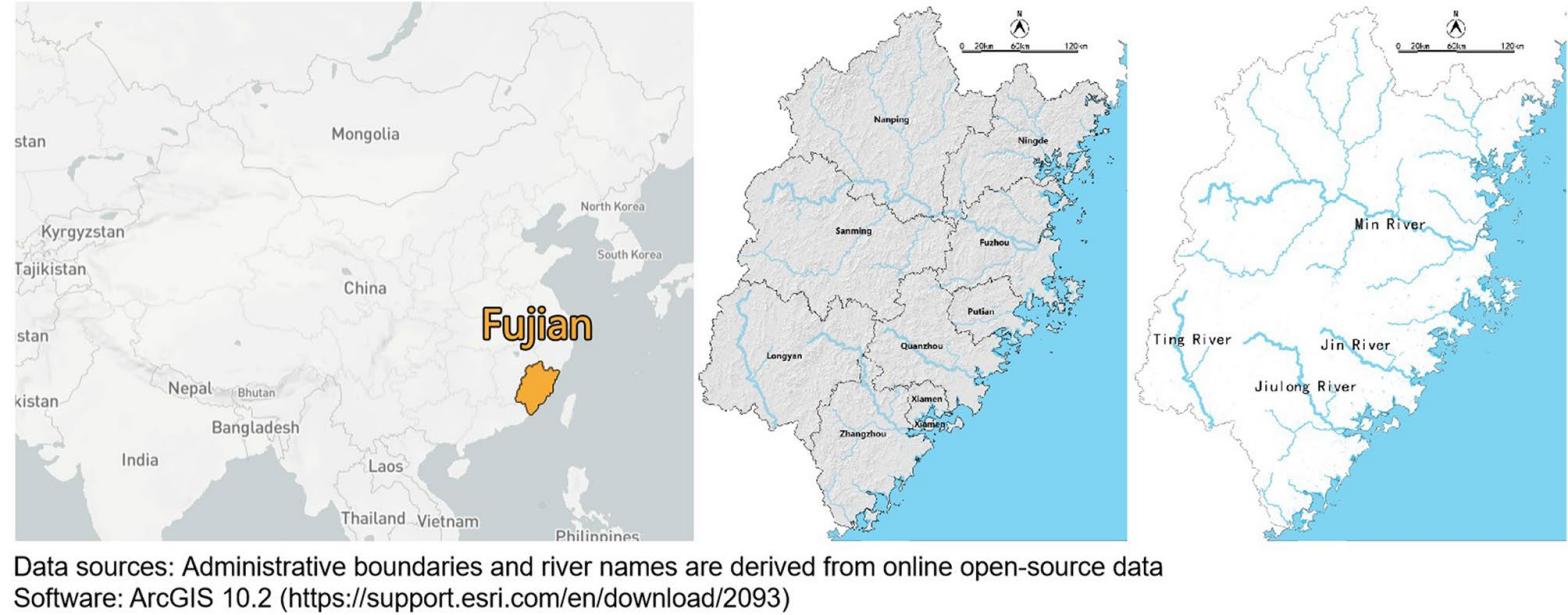
Location and map of Fujian Province, China.

Fujian province is located on the southeast coast of China. The province covers total land area of 121,400 km^2^. The terrain has a high elevation in the northwest and low in the southeast. Mountains and hills account for about 90% of the total area. Fujian includes five major rivers: Min River, Jin River, Jiulong River, and Ting River (Fig. 1).

### Materials and methods

Land use data are available from the Geospatial Date Cloud (https://www.gscloud.cn/sources/?cdataid=302&pdataid=10). DEM data for the study were downloaded from the Department of Geography, Tsinghua University (http://data.ess.tsinghua.edu.cn/).

**Figure 2:**
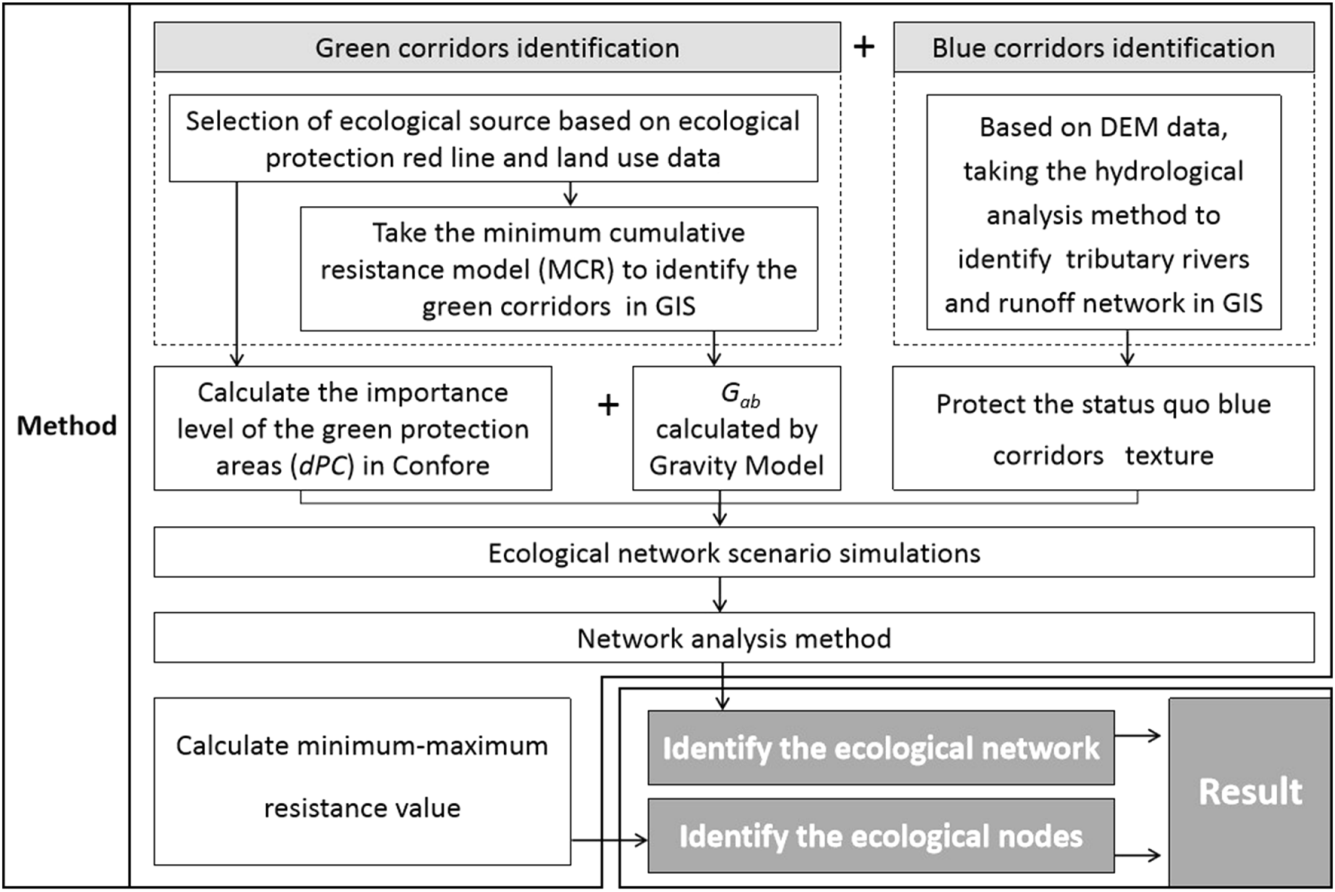
Visualisation of the research process.

As a first step in the analysis, ArcGIS was used to convert the required data into usable data. Selecting the green protection areas (GPA), the green corridors were identified based on the land use data. Blue corridors were identified and verified based on DEM data and existing hydrological data. Ecological network scenario simulations were constructed based on the importance level (*dPC*) value of GPA, the interaction force (*G*_*ab*_) between the GPAs, and the aim of protecting blue corridors. Finally, the ecological network structure was determined through network analysis. Simultaneously, ecological nodes were identified based on the ecological network and the minimum–maximum resistance value (Fig. 2).

### Obtaining a land use raster map

**Figure 3:**
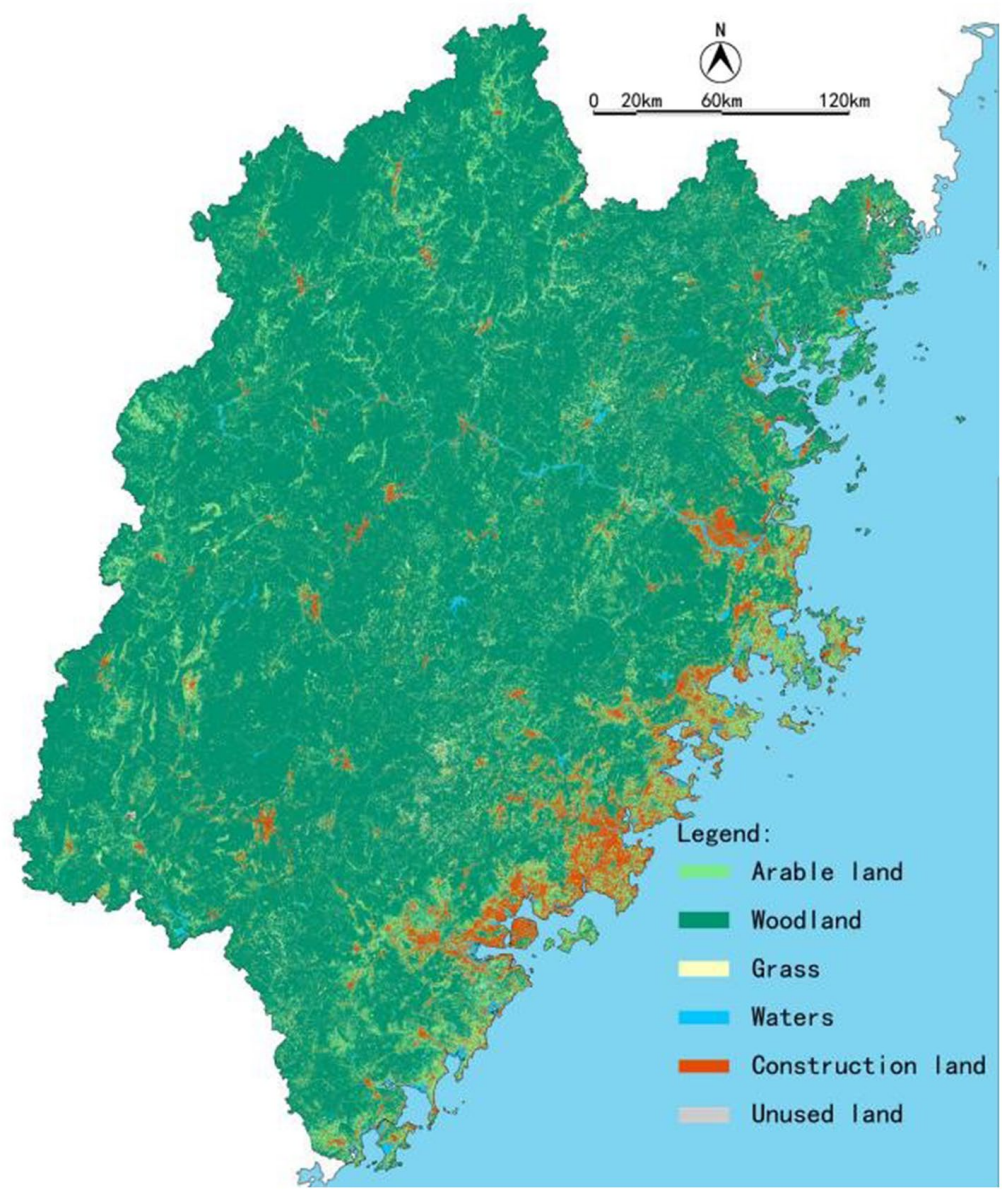
Land use status map for Fujian Province.

Splicing (Mosaic), cutting (Mask), reclassifying (Reclassify), and resampling (Resample) of the original land use data was undertaken to obtain the land use data of Fujian Province, in TIF format, for further analysis (Fig. 3).

### Minimum cumulative resistance model for green corridors

Minimum cumulative resistance model (MCR) is a model for calculating the minimum cumulative resistance from a grid-based map on which estimated dispersal resistances basic on landscape types^25,26,27^. The calculation formulas used are as follows:

1 $$V_{MCR} = f\min \sum\limits_{j = n}^{i = m} {(D_{ij} R_{i} )}$$

where *V*_*MCR*_ is the value of the minimum cumulative resistance; *f* is a positive correlation function between the minimum cumulative resistance and the ecological process; min denotes the minimum value of cumulative resistance produced in different processes of patch *i* transforming into a different patch *j*; *D*_*ij*_ is the spatial distance between patch *i* and patch *j*; and *R*_*i*_ represents the resistance value that exists in the ecological transition. The system of the resistance value will have a significant impact on the results of *V*_*MCR*_ and the same patch may have different resistance value for different ecological processes.

**Figure 4:**
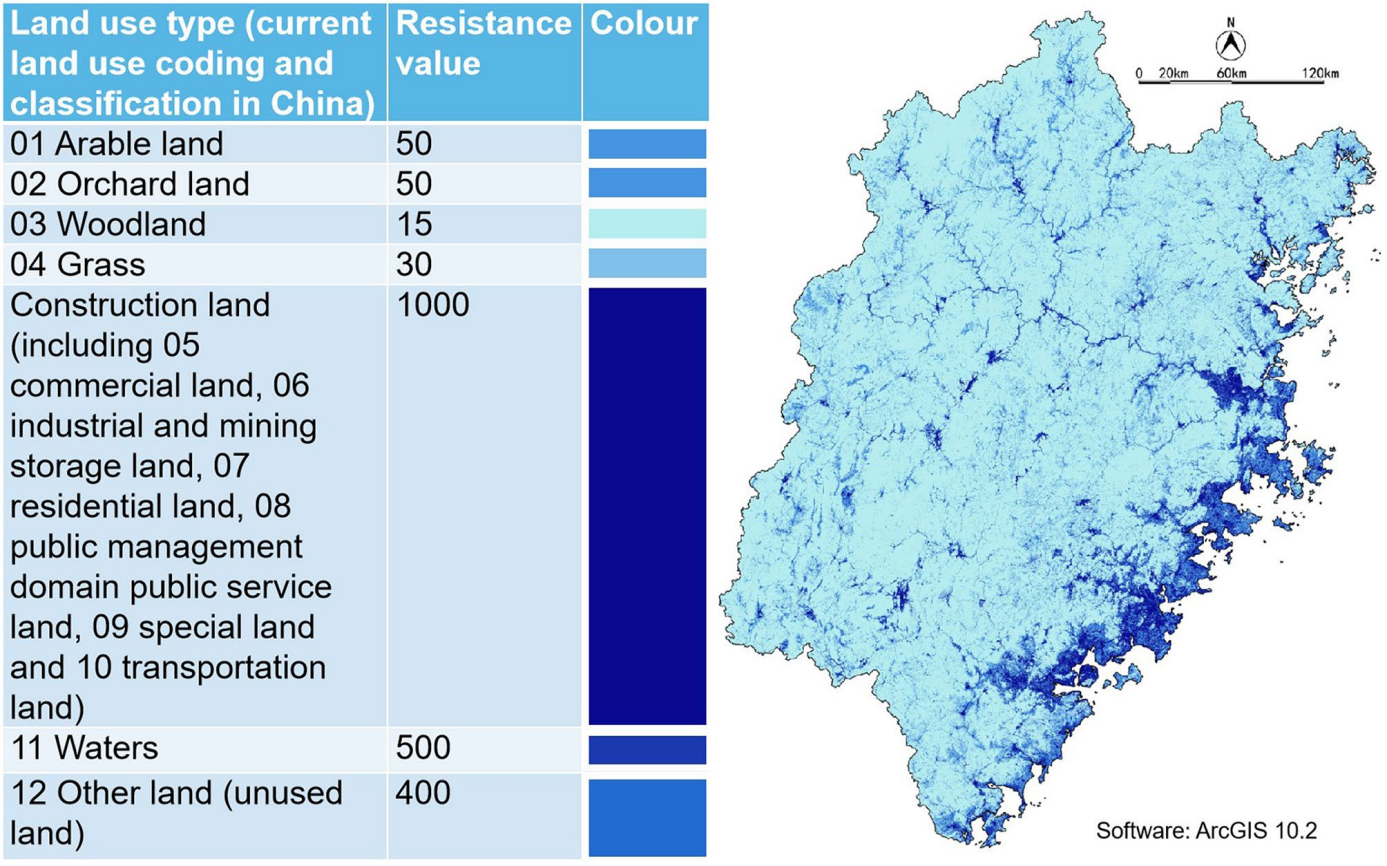
Resistance surface of land use in Fujian Province.

**Figure 5:**
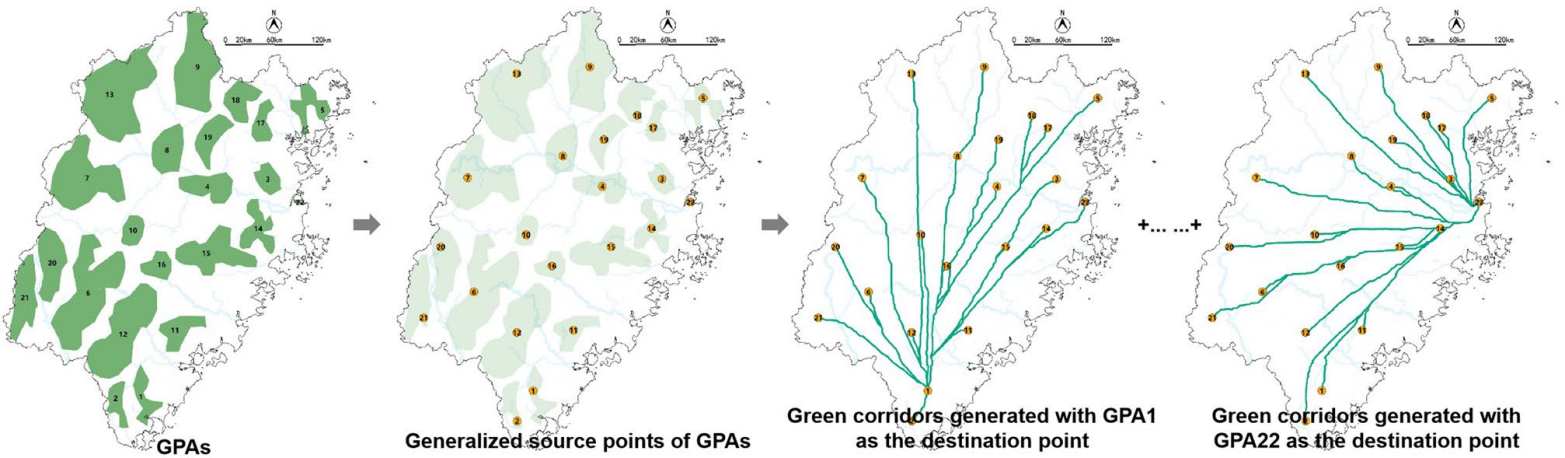
The generation process of green corridors in Fujian Province.

Referencing related studies^28–30^ and considering Fujian's actual situation, we determined the resistance value and took the land use type data to build the resistance surface in ArcGIS (Spatial Analysis—Raster Reclass—Reclassify), as shown in Fig. 4. Then, according to the generalized source points generated by ArcGIS and ecological data, manually adjusted the position of GPA source points and generated the green corridors through the command of Spatial Analyst-Distance (Cost Distance and Cost Path) in ArcMap (Fig. 5).

### Hydrological analysis methods for blue corridors

**Figure 6:**
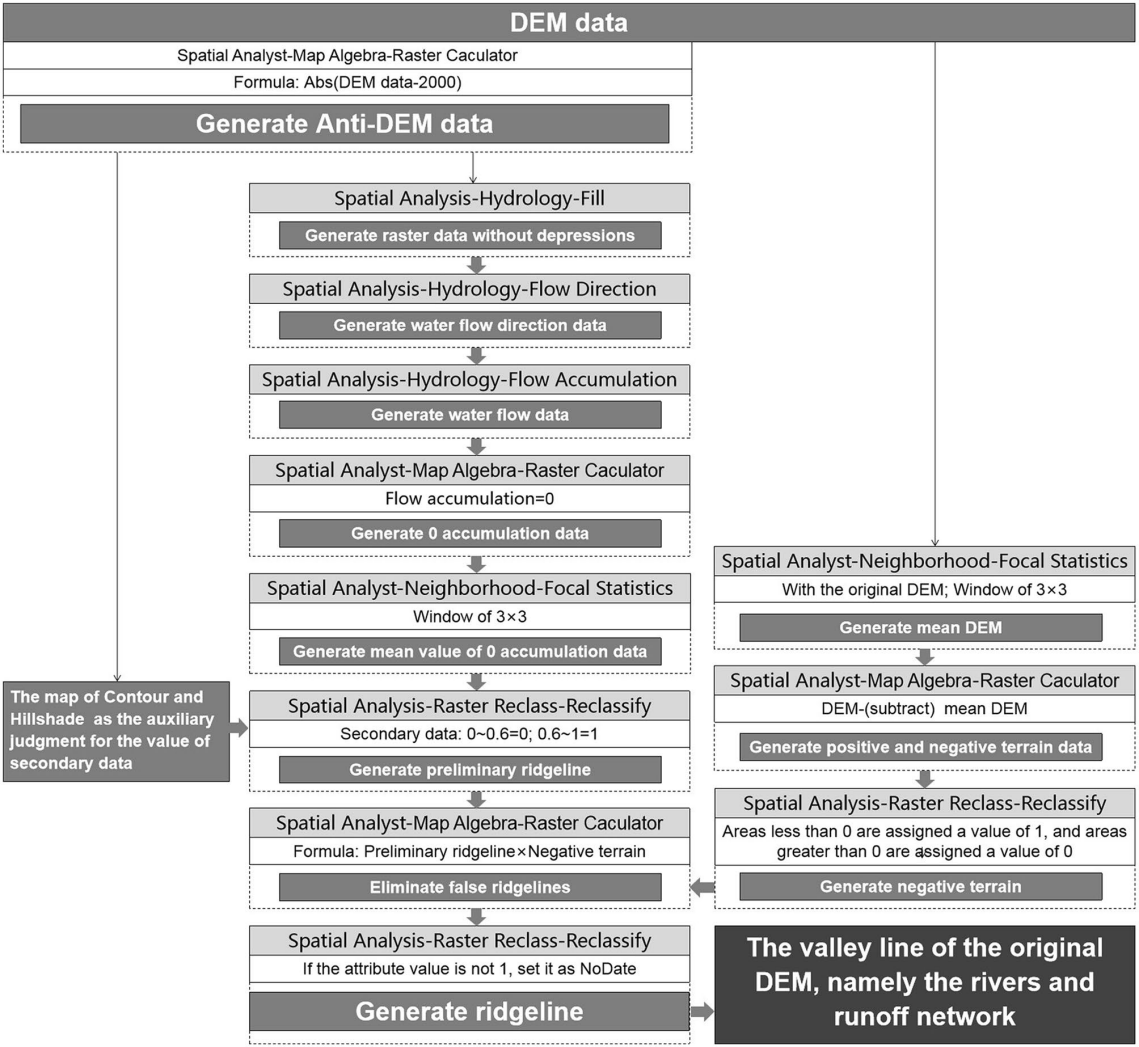
Process diagram for identification of Fujian’s rivers and runoff network by ArcGIS.

**Figure 7:**
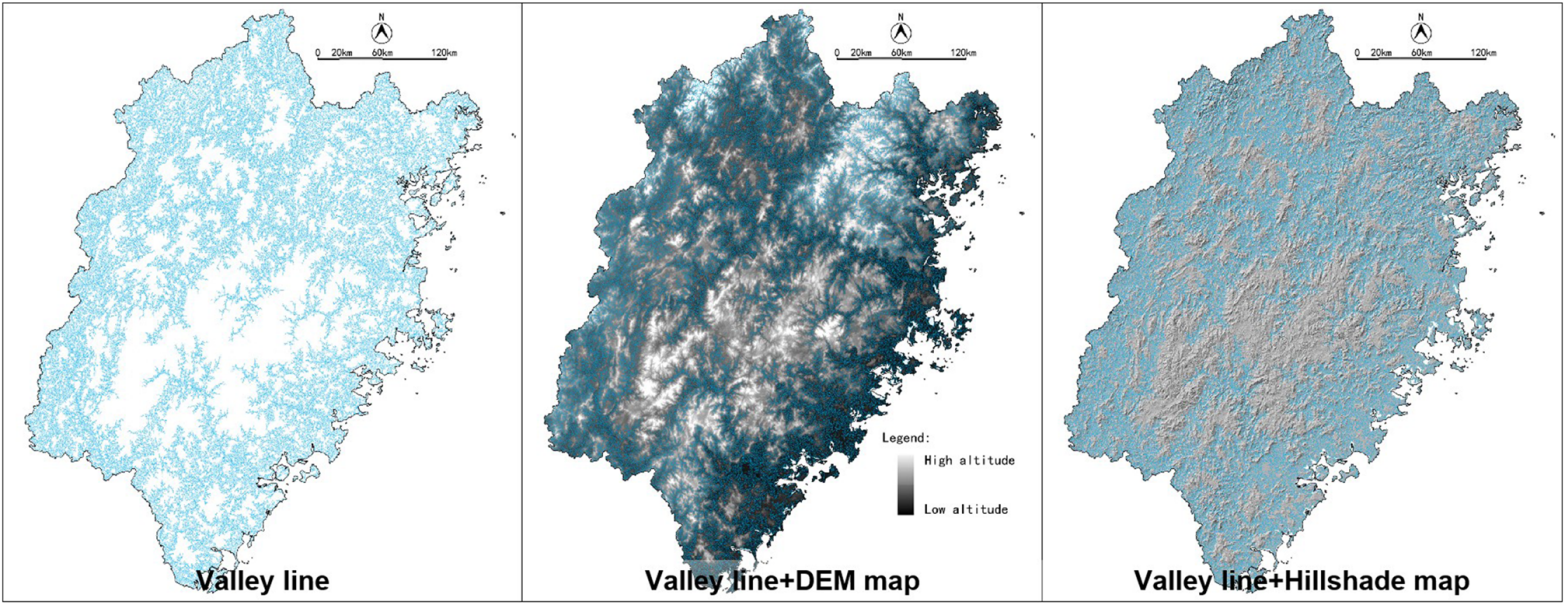
Valley line map of Fujian Province created by ArcGIS 10.2.

Based on the DEM data, with the Spatial Analyst-Hydrology tool of ArcGIS 10.2, the valley line within the research area could be identified (Fig. 6). Additionally, combining with the map of Hillshade and DEM, identify tributaries through manual selection (Fig. 7). The valley line map is the base of the blue corridors.

### Gravity model for the selection of ecological candidate network

**Figure 8:**
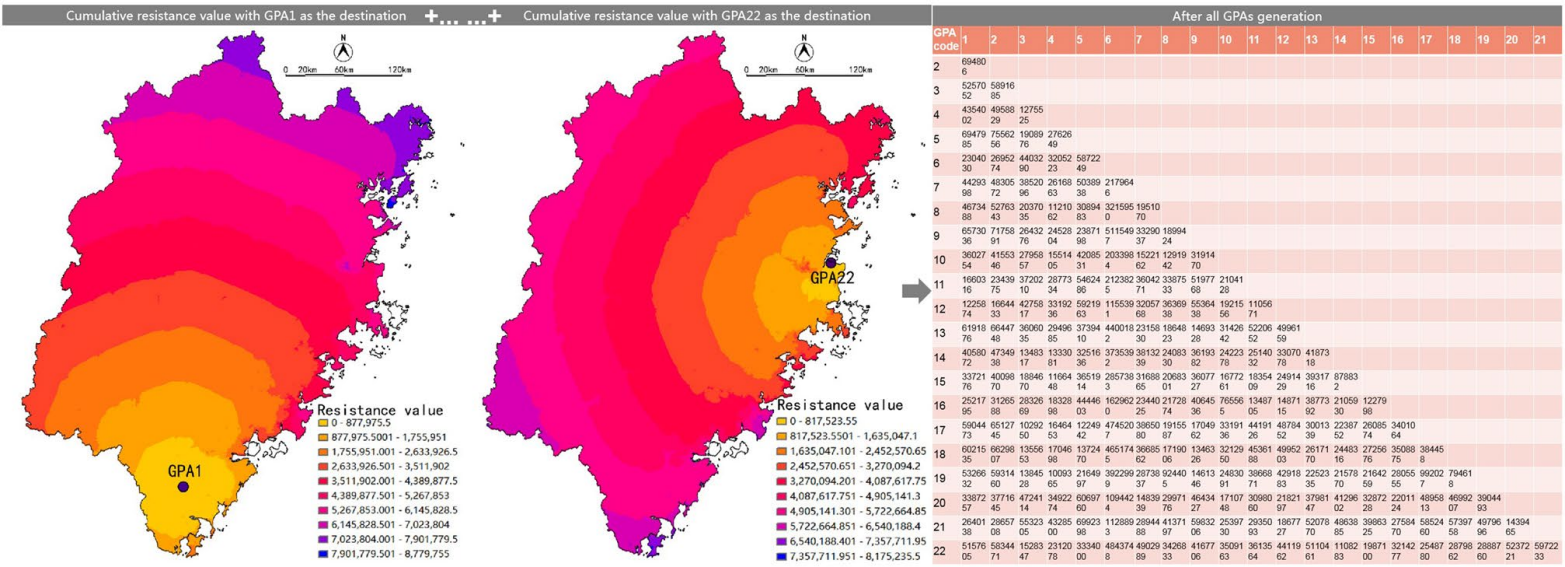
Schematic diagram of *L*_*ab*_.

In ecological planning, gravity modelling is usually used to measure the potential value of ecological process between protected land ^30–33^. We selected the improved gravity model formula of Kong and Yin^31^, as shown below:

$$G_{ab} = \frac{{L_{\max }^{2} \ln (S_{a} S_{b} )}}{{L_{ab}^{2} P_{a} P_{b} }}$$

where *G*_*ab*_ is the interaction force between GPA *a* and *b*, *P*_*a*_ and *P*_*b*_ are the resistance values of GPA *a* and *b*, *S*_*a*_ and *S*_*b*_ are the areas of GPA *a* and *b*, *L*_*ab*_ is the cumulative resistance value of the corridor between GPA *a* and *b*, and *L*_*max*_ is the maximum resistance of all the corridors in the study area. *L*_*ab*_ and *L*_*max*_ can be calculated by ArcGIS (Fig. 8).

### Classification method of the GPAs

**Figure 9:**
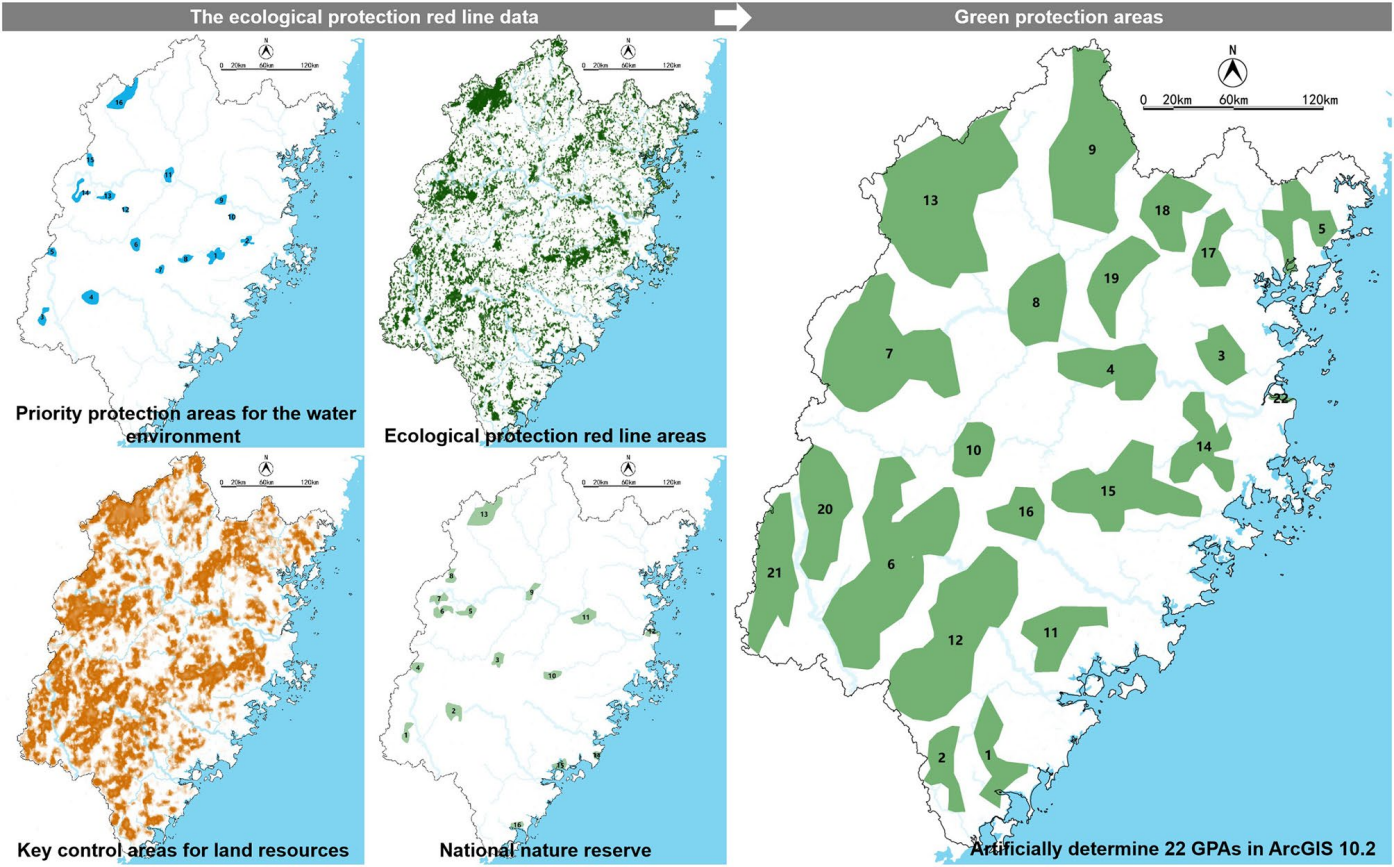
Process diagram of GPA determination in Fujian Province.

The ecological protection red line data, which contains priority protection areas for the water environment, ecological protection red line areas, key control areas for land resources, and national nature reserve, is the main basis for determining GPAs. Artificially re-evaluating areas inside the ecological protection red line, we artificially determined 22 GPAs in ArcGIS 10.2 (Fig. 9).

Considering the importance of connectivity^34,35^, we used the probability of connectivity metric (*PC*) and the importance level (*dPC*) to classify the GPA^36,37^. The calculation formulas were as follows:

3 $$PC = \frac{{\sum_{i = 1}^{n} {\sum_{j = 1}^{n} {p_{ij} a_{i} a_{j} } } }}{{A^{2} }}$$

4 $$dPC = \frac{{PC - PC_{remove} }}{PC} \times 100\%$$

where *PC* is a graph-based availability metric that quantifies functional connectivity, 0 ≤ *PC* ≤ 1, the larger the *PC* value is, the higher the connectivity degree of the GPA is; n represents the total number of GPA in the city area; *P*_*ij*_ is the maximum product of all path probabilities between GPA *i* and GPA *j; a*_*i*_ and *a*_*j*_ are the areas of GPA *i* and *j*; *A* is the total area of the city area; *dPC* is the change (in %) of the connectivity index after removing one GPA, it represents the importance level of one GPA; *PC*_*remove*_ is the overall index value of the remaining GPA after removing a single GPA.

Using Conefor2.6 to realize the calculation process, the parameter settings were as follows:

1. Calculate the distance between all features.
2. The Distance of Probabilistic indices was 2000 m.
3. The corresponding probability was 0.5.

### Network analysis for the identification of the ecological network

**Figure 10:**
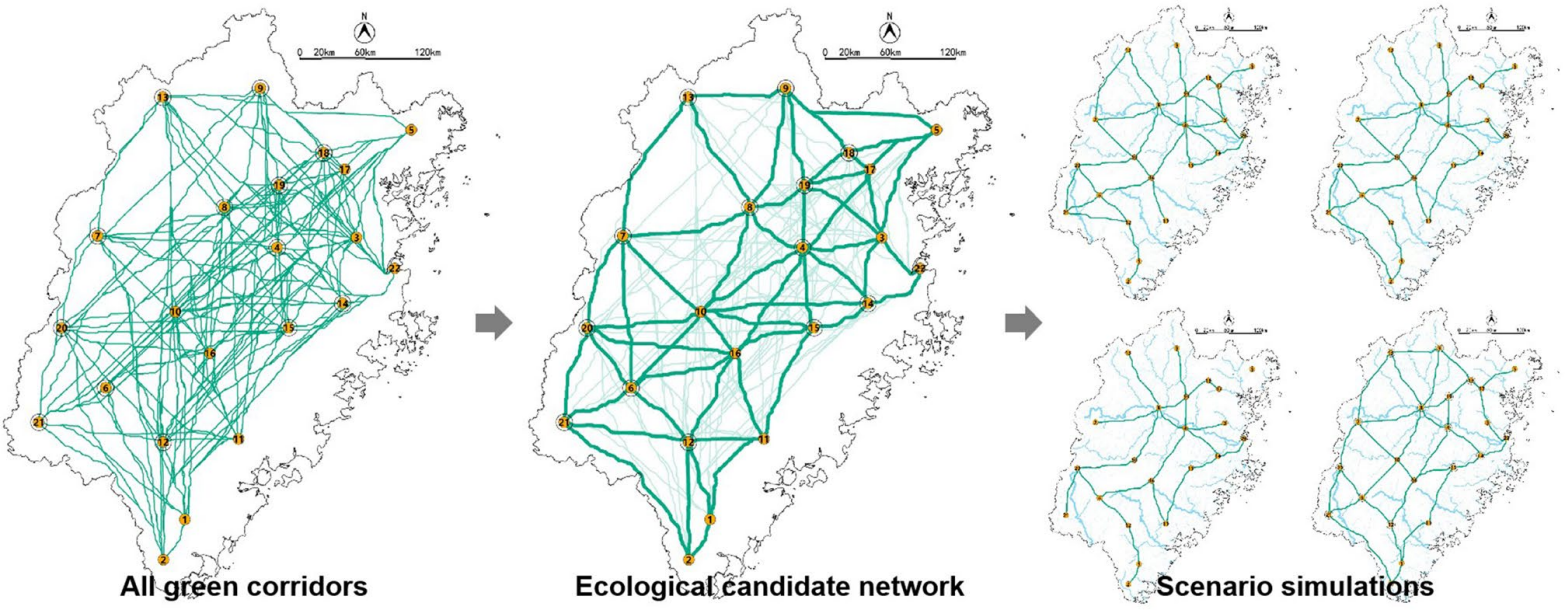
The process diagram of scenario simulations.

Selecting the high value of *dPC* and *G*_*ab*_ of GPA, we selected an ecological candidate network within the green corridors generated. Next, simulated scenarios network were generated within the ecological candidate network with the aim of blue corridors protection (Fig. 10). The network analysis method^32,38–42^ was introduced to evaluate the scenarios. The indexes calculation formula as follows:

5 $$\alpha = \frac{l - v + 1}{{2v - 5}}$$

where *a* is the loop index, the number of loops present divided by the maximum number of loops possible; *l* is the number of corridors, and *v *is the number of GPA..

6 $$\beta = \frac{l}{v}$$

where *β* is the average connection index, if *β* < 1, there is a dendrogram that occurs; if *β* = 1, there is a single circuit; and if *β* > 1, it means more complex levels of connectivity exist.

7 $$\gamma = \frac{l}{{l_{\max } }} = \frac{l}{3(v - 2)}$$

where *λ* is the network connectivity index, the ratio of the number of links in a network to the maximum number of links possible.

8 $$CR = 1 - \frac{l}{d}$$

where *CR* is the cost ration and reflects the network's effectiveness, *d* is the accumulative resistance of the corridors calculated according to resistance value by using ArcGIS.

### Identification of ecological nodes

**Figure 11:**
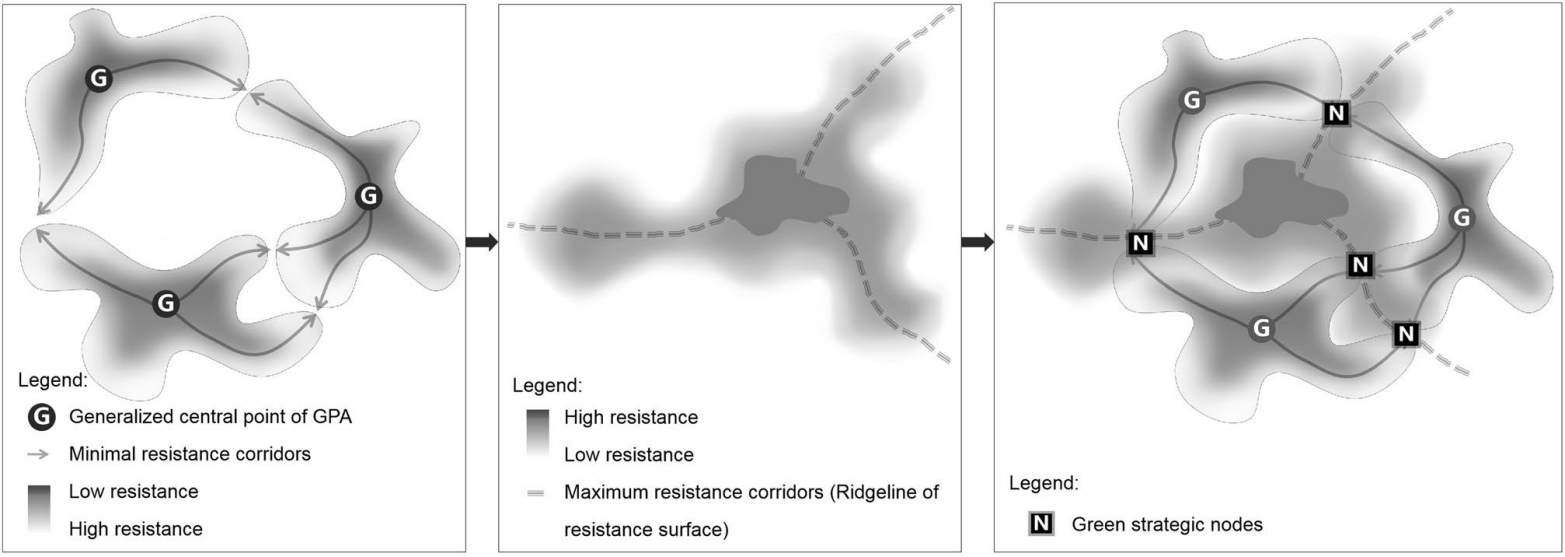
Recognition methods of the minimum–maximum value nodes.

In this research, the minimum–maximum node was taken as the ecological node, which is in the saddle formed at the tangent part of the equivalent resistance line centered on GPA^43–45^. It represents the minimum–maximum value on the resistance surface, which can be regarded as an area weak in ecological function. The identification method was as shown in Fig. 11.

**Figure 12:**
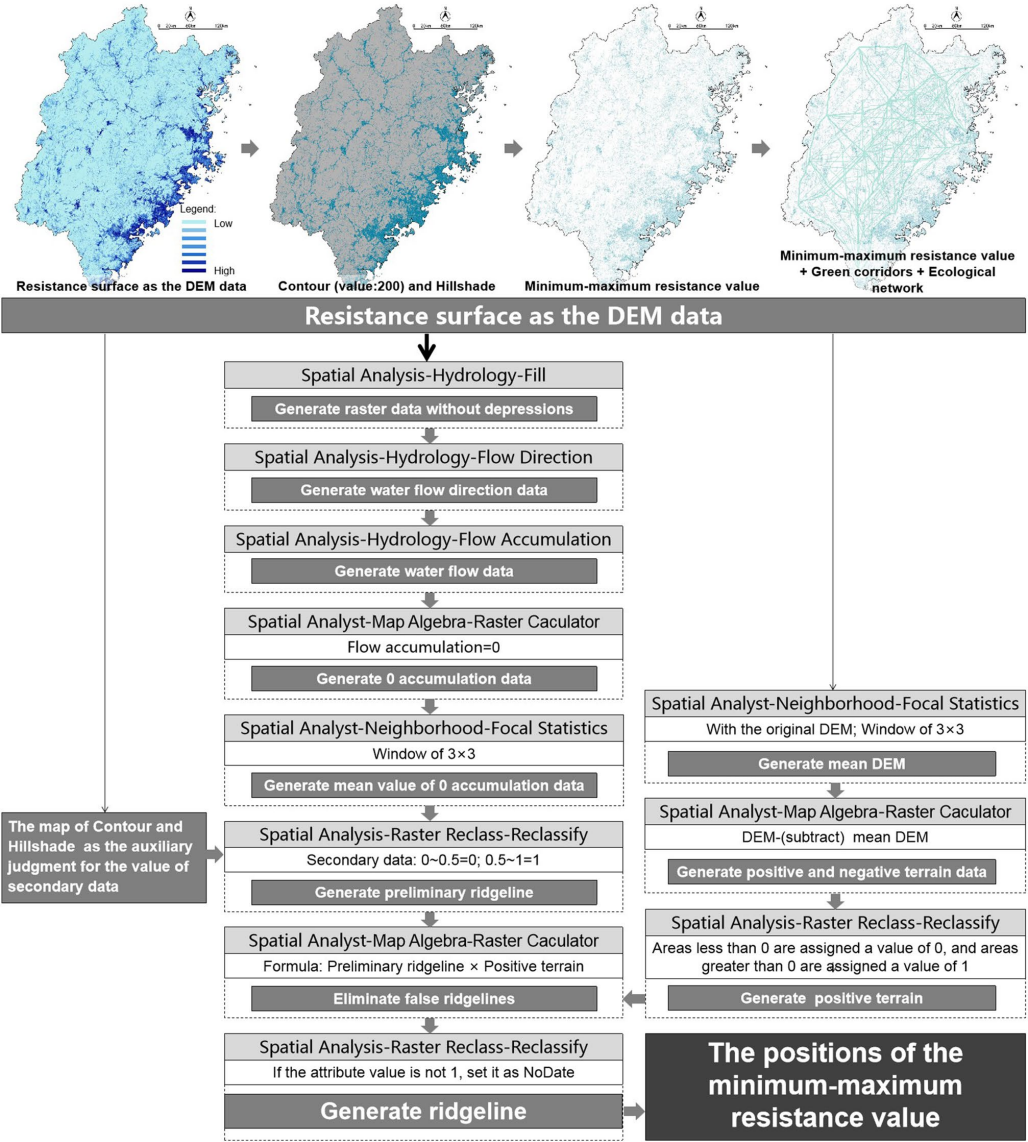
Diagram presenting the identification of ecological nodes in Fujian Province.

For the Fujian research, taking the resistance surface as the DEM data, we identified the minimum–maximum value by the method of hydrological analysis, which was a method to identify the ridgeline, as shown in Fig. 12.

## Results

### Identification of the green corridors

**Figure 13:**
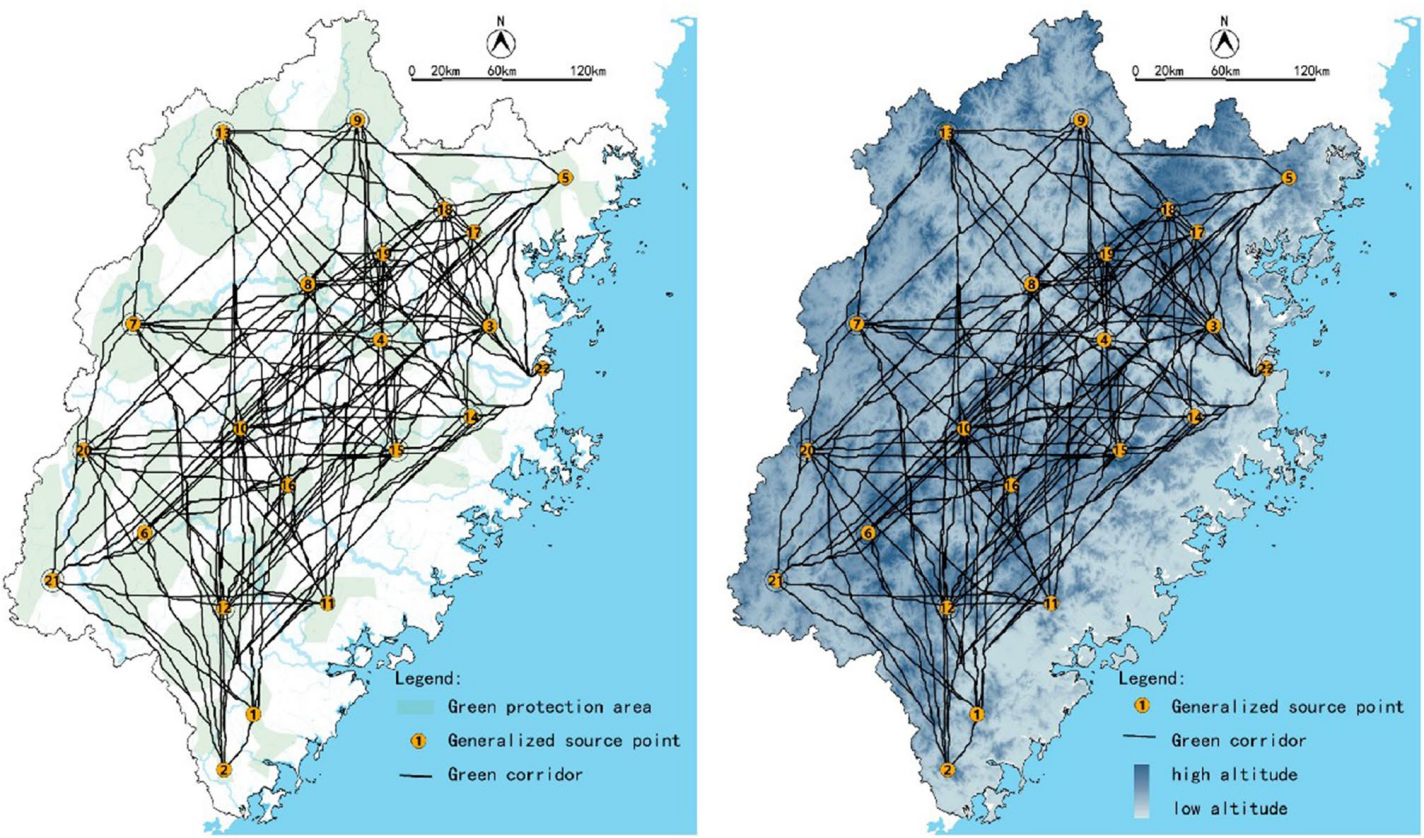
The green (minimum resistance) corridors of the Fujian Province.

Based on the assistance value system, we identify green corridors by the ArcGIS 10.2 software. As Fig. 13 shows, the green corridors of Fujian are mostly located in the two mountain ranges extended from northeast to southwest. The mountain areas are at the intersection of multiple corridors and play an essential role in ecological connection.

### Identification of the blue corridors

**Figure 14:**
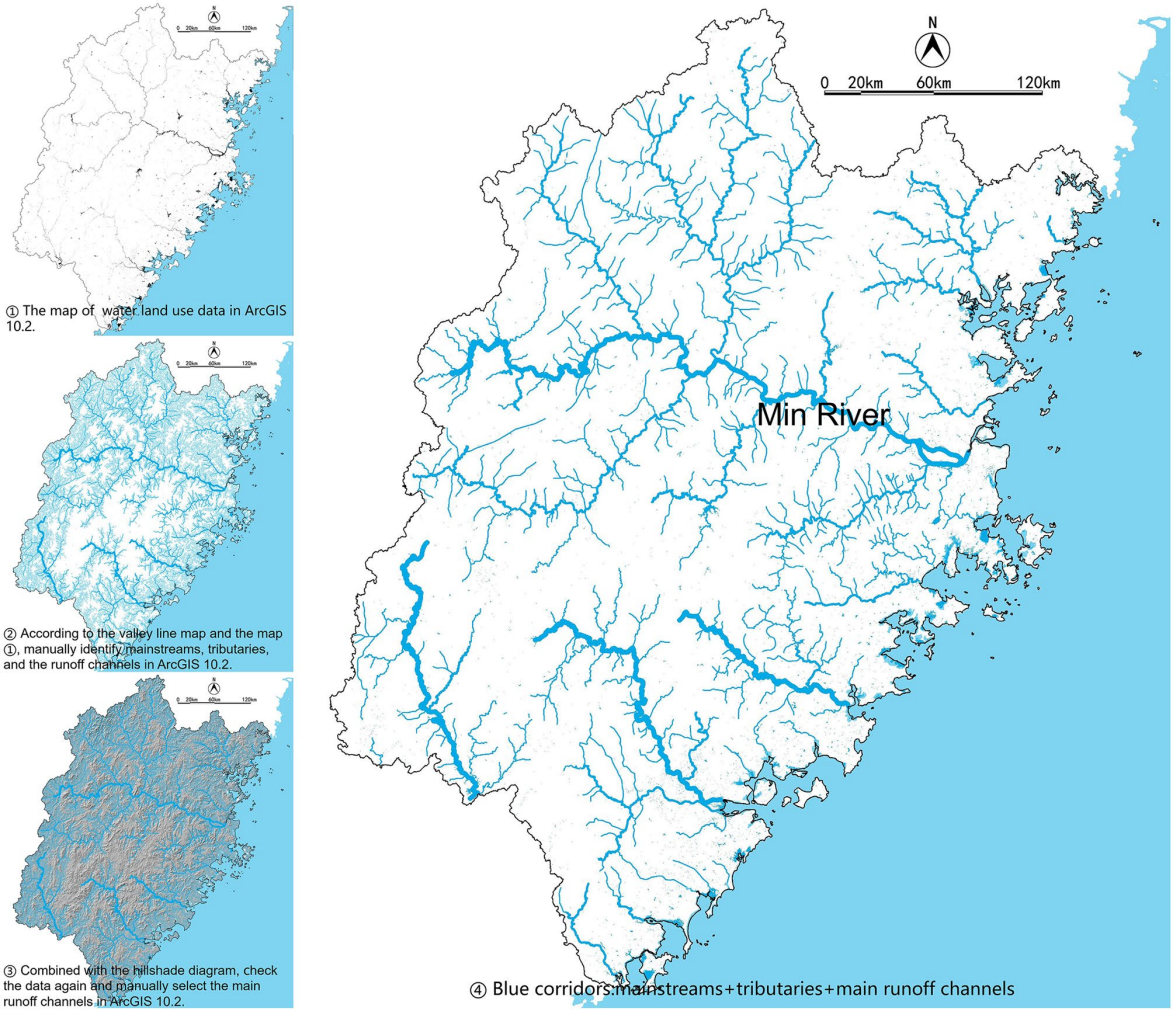
Blue corridors of the Fujian Province.

Combining the valley line map (Fig. 7) with the land use data of water, a total of 4 mainstreams were identified in ArcGIS 10.2. Each mainstream has several tributaries. As the longest mainstream in Fujian Province, Min River’s catchment area encompasses most of the scope of the north and central region, with the most tributaries number of eight. The northwest area belongs to the upper river of the Min River, whose water conservation capacity has an essential impact on the flood control of the downstream area of Fuzhou. In addition, runoff channels that also be identified. By comparing topographic maps, we selected some of the main runoff channels, which combine with the mainstreams and tributaries to form the Fujian blue corridors, as Fig. 14 shows.

### Determination of ecological candidate network

**Figure 15:**
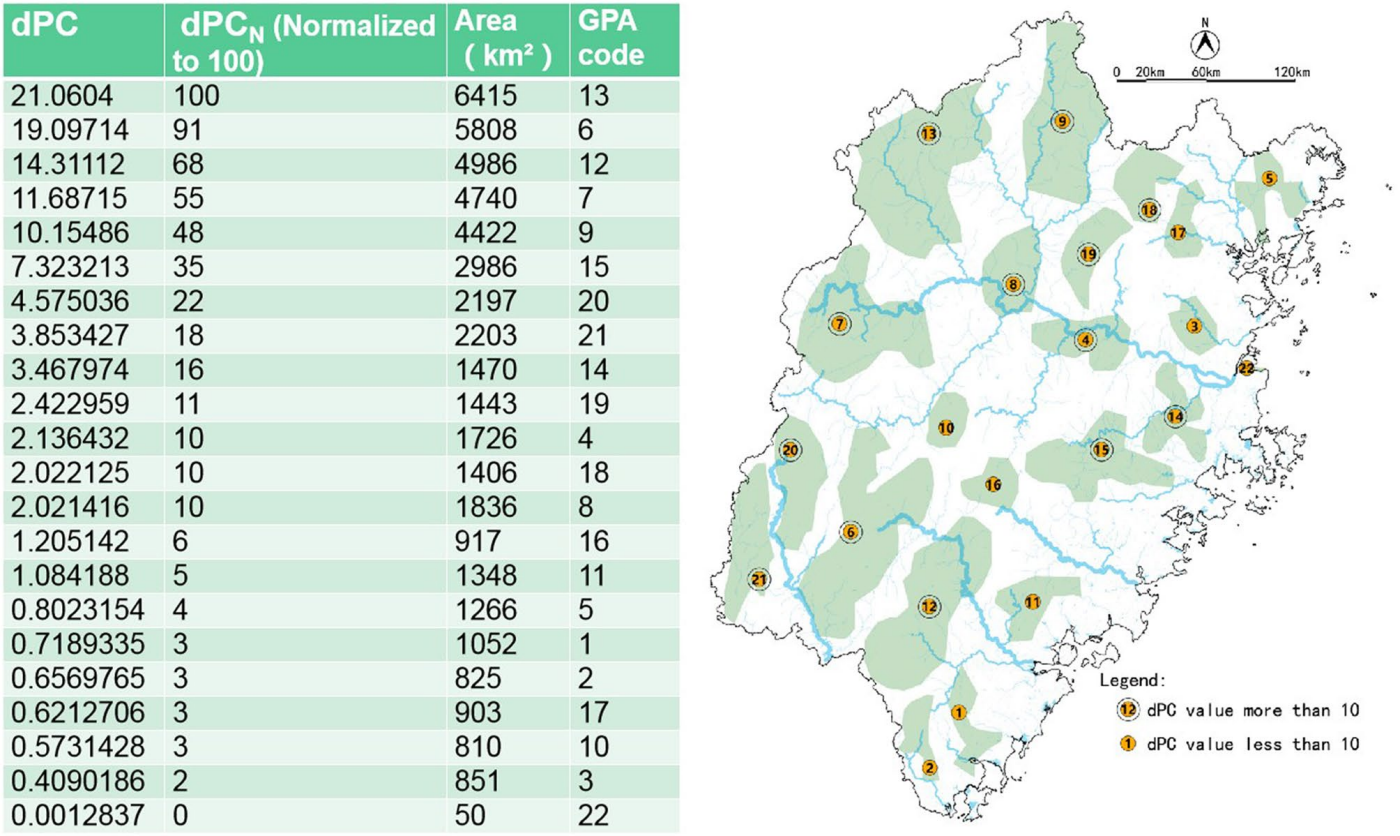
The *dPC* value of GPAs in Fujian Province.

**Figure 16:**
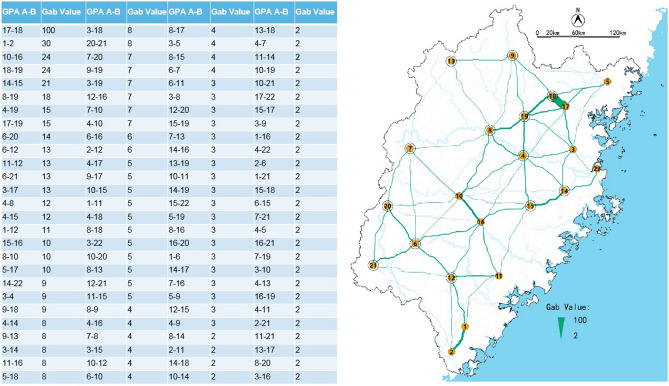
Ecological candidate networks in Fujian Province.

To facilitate the comparison, we normalized the *dPC* and *G*_*ab*_ results to 100. The ecological candidate network selected 13 GPAs, whose *dPC* values were more than 10, as shown in Fig. 15. There are 108 green corridors whose *G*_*ab*_ values were higher than 2, were selected into ecological candidate networks, as shown in Fig. 16.

### Selection of ecological network

We prioritized the corridors with high *dPC* value and high *G*_*ab*_ value into the candidate ecological network, and the final ecological network is selected from the candidate networks (Fig. 16).

**Figure 17:**
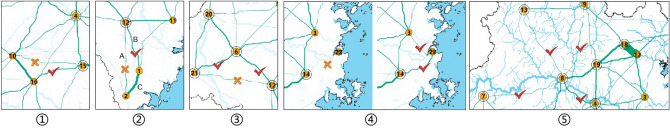
Diagram of principles for ecological network selection.

To reduce the number of corridors and maximize the efficiency of the network, the selection of ecological network corridors mainly follows the following principles (Fig. 17):

1. When there are multiple parallel corridors around the adjacent distance area in the candidate network, we only selected one corridor and eliminated other corridors.
2. When corridor A between the two GPAs is similar to the sum of the two corridors BC constructed by them and the other GPA, we settle corridors BC instead of corridor A.
3. Priority to retain corridors with higher *G*_*ab*_ values.
4. All GPAs have at least one corridor connected to other GPAs.
5. Preferentially include the corridors which paralleled the mainstream or located in the dense blue corridor areas.

**Figure 18:**
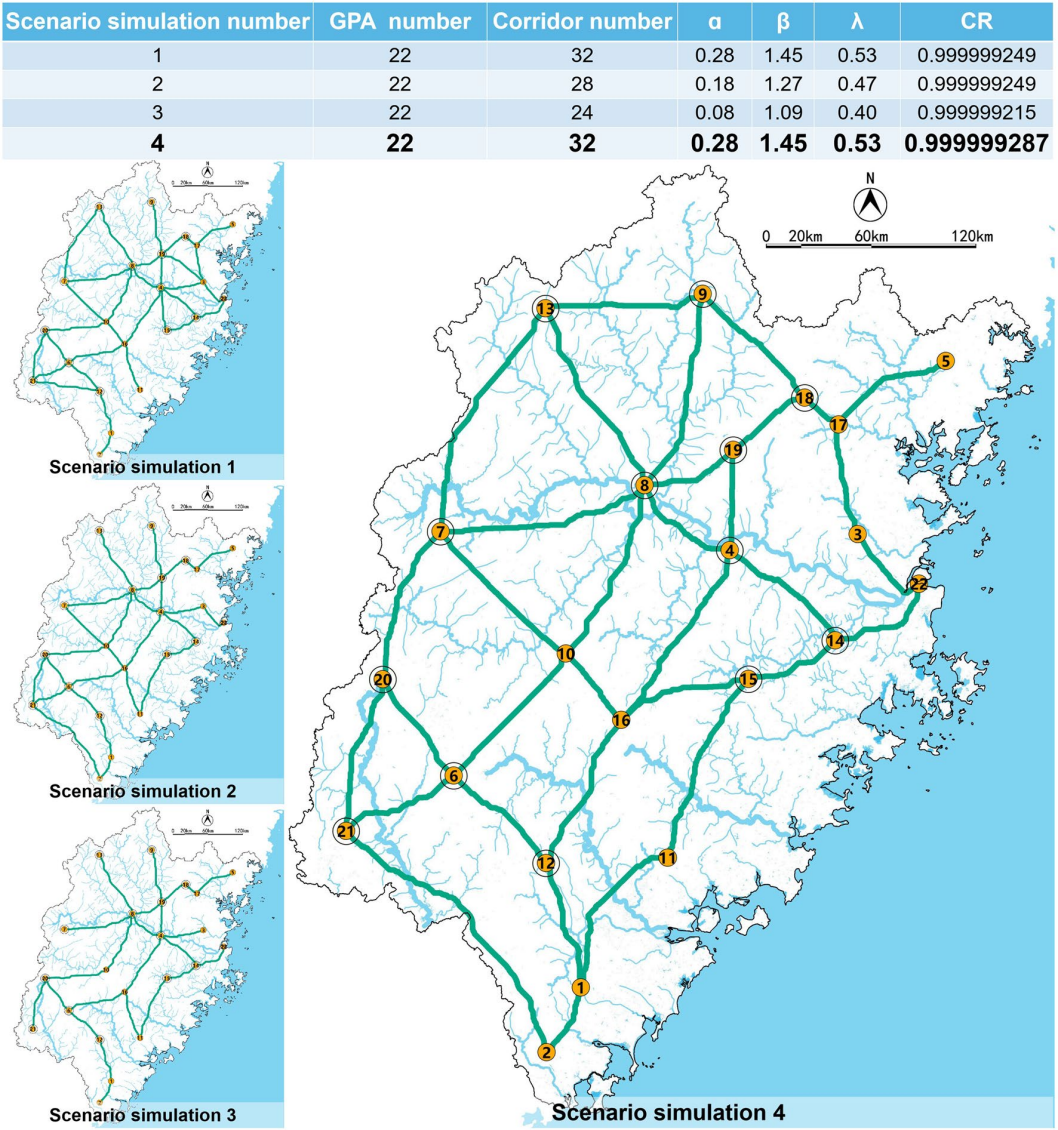
Network analysis results of four scenarios and ecological network of Fujian Province.

In addition, we hope that the composition modes of the four scenarios are as different as possible, and the number of connected corridors is as diverse as possible. Thus, we simulated four network connection modes, compared their network analysis results, and then took scenario 4 as the final ecological network, as shown in Fig. 18.

### Identification of ecological nodes

**Figure 19:**
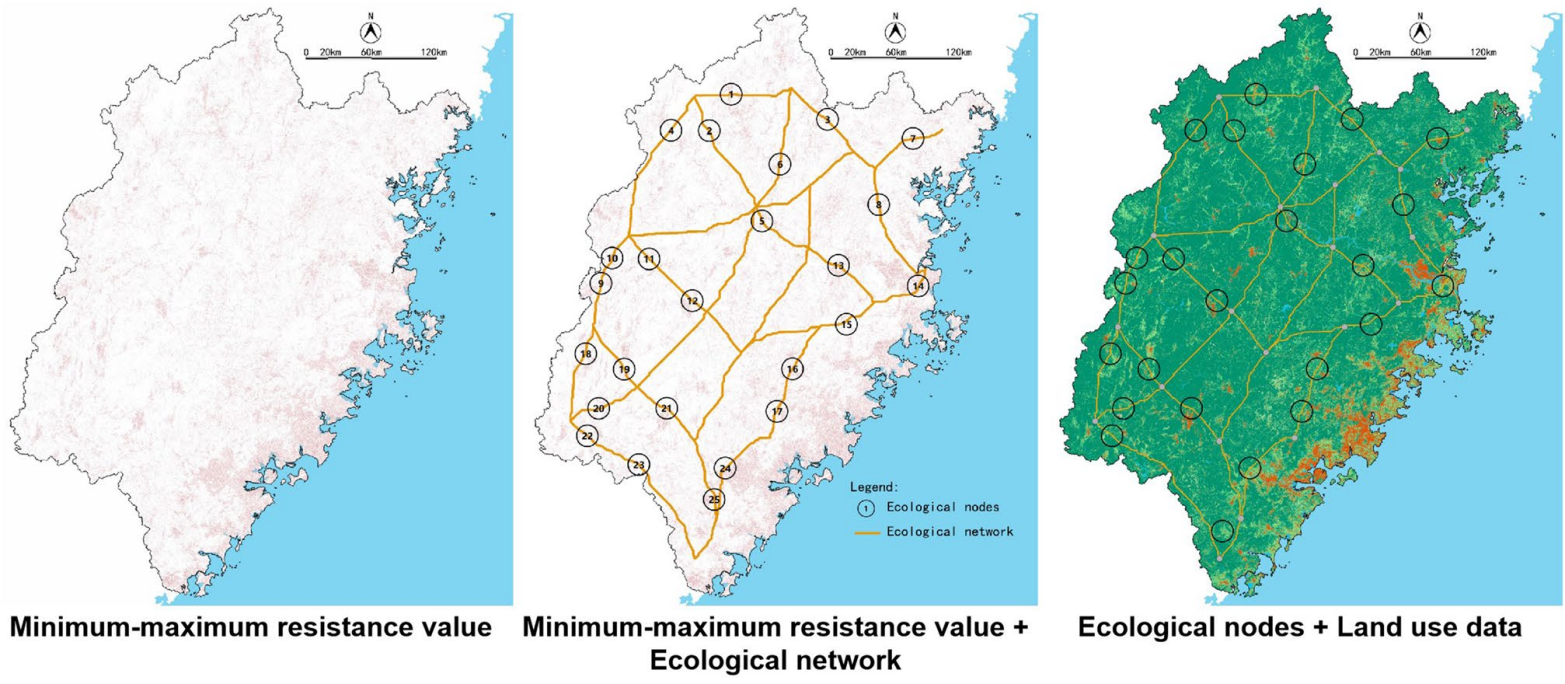
Ecological nodes of Fujian Province.

As shown in Fig. 19, by superimposing the minimum–maximum resistance value results and the final ecological network, we selected 24 ecological nodes where the minimum–maximum resistance and the minimum resistance overlap. By checking the current land use and status quo, 24 ecological nodes were dominated by construction land and had multiple land-use types. All the nodes have a high degree of land fragmentation, which was in line with weak environmental features in the ecological nodes.

## Discussion

Since the reform of China's urban and rural planning in 2018, green land has replaced the construction land as the dominant fact of the urban layout. This paper introduces the ecological environment planning method into the urban and rural planning field to adapt to the changes. Based on the land use data, we identified the green corridors. Additionally, the blue corridors were identified by the DEM data in ArcGIS. Since both land use data and DEM data are open-source data, the planning method adopted in this article has achieved barrier-free data sources. The network analysis method further realizes the identification of ecological networks quantitatively. With the help of the minimum–maximum resistance values, ecological nodes had also been identified. These identification methods all provide a specific reference for the GSSP toward the direction of quantitative planning.

### Suggestions for the ecological network of Fujian

**Figure 20:**
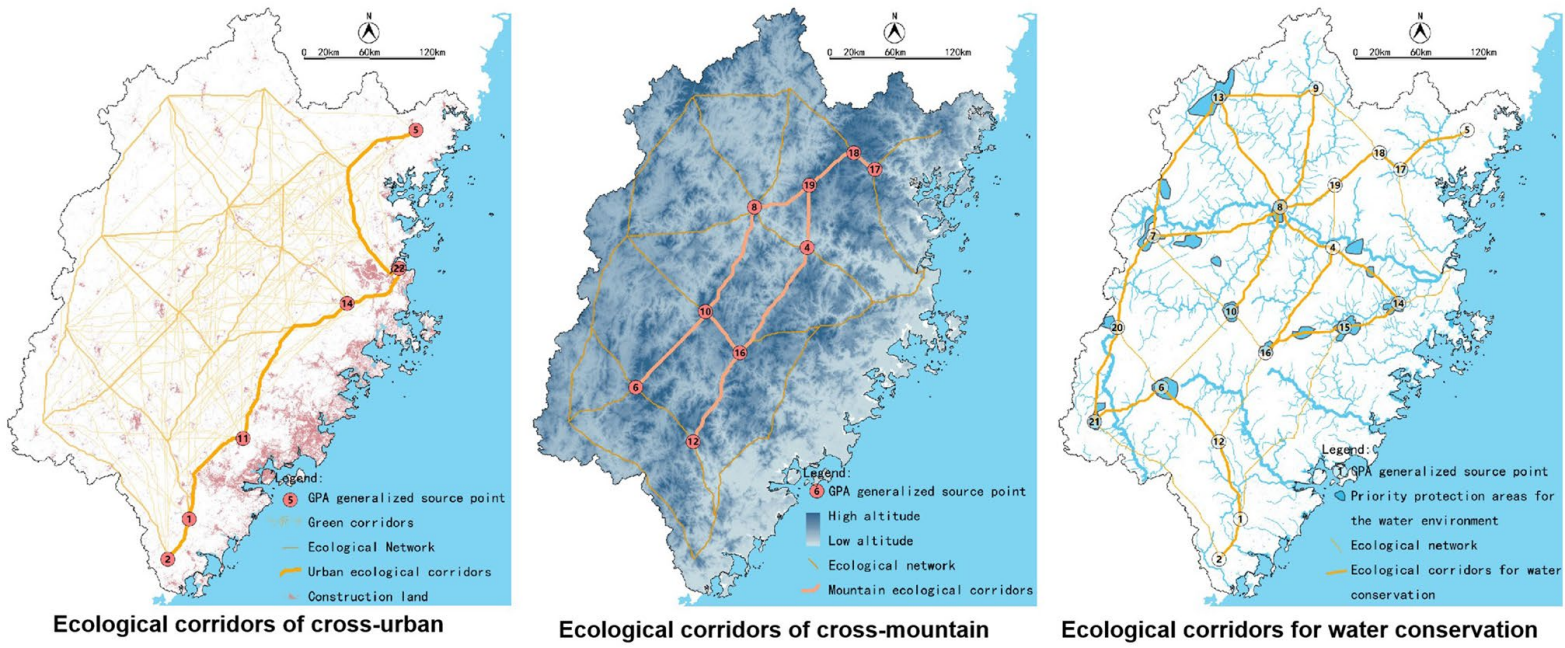
Three types of ecological corridors.

Based on our analysis, suggestions can be made for the delineation and development of the ecological network of Fujian. The ecological network identified in Fujian Province can be divided into three types: cross-urban type, cross-mountain type and water conservation type, as shown in Fig. 20.

In the cross-urban ecological network, there are areas suitable for the ecological process of small mammals and birds. Simultaneously, combining with construction land, the functions of noise reduction, wind protection, and environmental purification can be added. Additionally, this part of the network is also the primary carrier for the construction of inter-city sightseeing corridors.

The cross-mountain ecological corridors are suitable for creating biological migration corridors, such as migration corridors for medium and large mammals and birds, which can assist in the construction of natural habitats, focusing on protection and restoration native ecological environment.

Ecological corridors of water conservation connect the priority ‘blue’ protection areas. This type of corridors can not only be used to conserve water sources and ensure water quality but also can be equipped with green storage facilities to reduce the pressure of flood discharge in the downstream catchment basin. In terms of Corridor 16-15-14, although the blue network parallel to it is not the mainstream, it has a dense tributary and runoff network in this area. It is located upstream of the Fuzhou urban area and has crucial impacts on water quality and flood discharge and should be strictly protected. Corridor 8-9 and 8-13 are located in dense blue network areas, which play an essential role in the blue network's ecology and should also be equipped with some green storage facilities.

### Suggestions for the ecological nodes of Fujian

**Figure 21:**
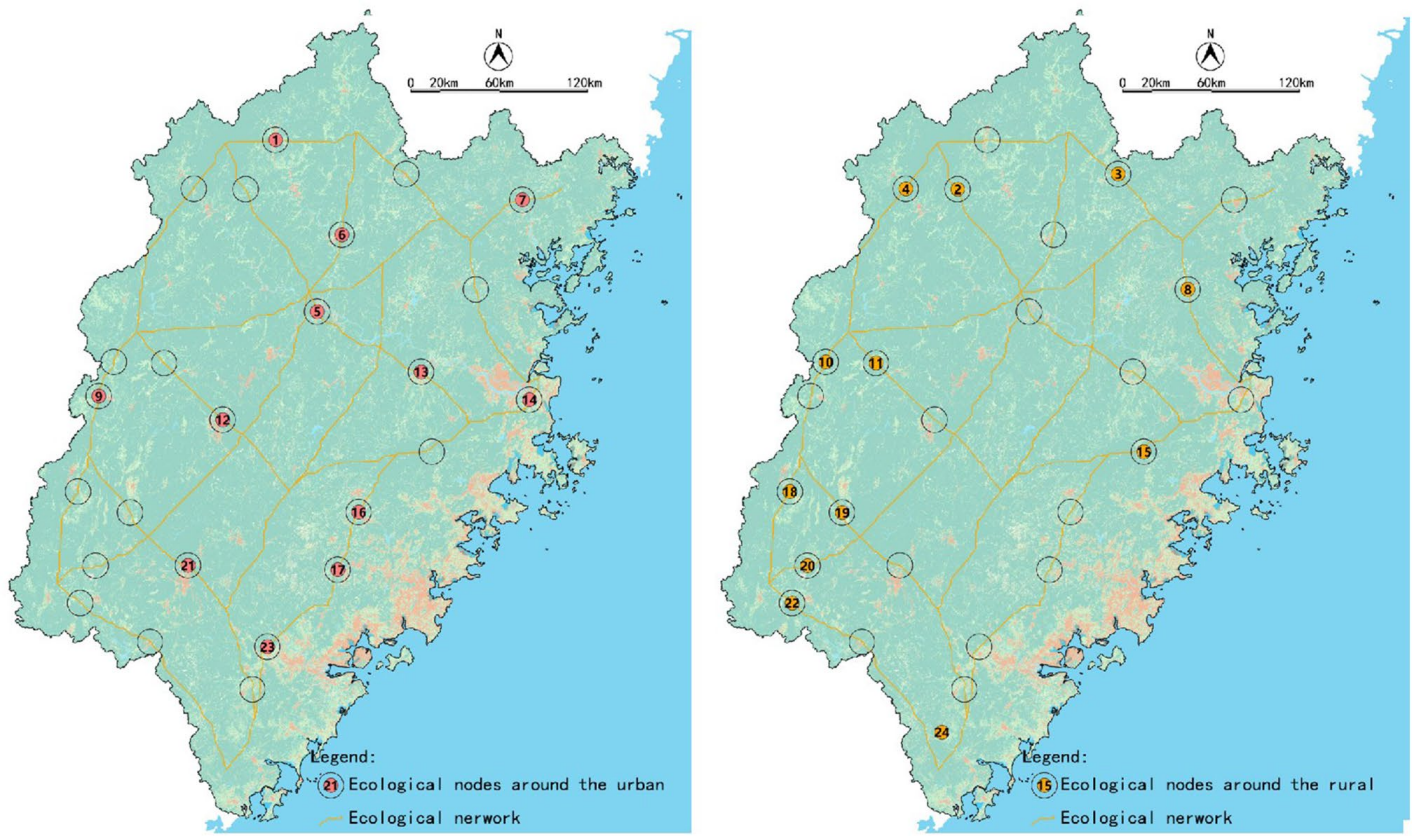
Two types of ecological network nodes, Fujian Province.

The ecological nodes of Fujian can be divided into two categories, which are of urban and rural types (Fig. 21). For urban ecological nodes, to ensure the quantity of green space in the construction land and convert other construction lands into the green land, which is a kind of construction land in China, can establish an ecological connection between construction land and non-construction land. It would be best to convert construction land into non-construction lands such as woodland and orchard if conditions permit. For the rural ecological nodes, strictly controlling the number of homesteads and restoring abandoned land to ecological land are the primary measures. It is also necessary to prevent land fragmentation and ensure the size of landscape patches.

### Restrictions of large-scale data on results

Unlike small-scale ecological planning^12,46,47^ as a planning method based on the large-scale data, provincial ecological network and node identification is mainly used to guide macro-planning and provide guidance for further detailed planning and design. The identification results need to be highly generalized and combined with manual selection. For example, the ecological nodes that are initially identified are vast in area, large in number, and scattered. As in the research of Morandi et al.^48^, Santos et al.^49^, and Dai et al.^50^, it is also necessary to assist in land use data, field conditions, landscape ecology, and other potentially relevant ecological environments to select the most suitable and reasonable area and determine the final ecological network and nodes.

### Open-source DEM data for blue corridors identification

Many studies have confirmed the critical role of water networks on land management and ecological functions^51–53^. Besides, the water network's influence in a small area on the ecological network and their mutual combination have also been studied^54,55^. However, the general lack of large-scale hydrological data has made few studies on combining large-scale ecological networks and water networks. Research results showed that in areas lacking hydrological data, blue corridors could be identified through the open-source DEM data. This method of identification also provides more possibilities for further collaborative research on blue and green networks.

### Expansion of ecological network functions

In the presented study, the ecological network was generated based on the structure of the green–blue network. The ecological network was taken as a crucial carrier for the multiple functions of the green space system. Many studies have pointed out that it consideration should be made of important functions such as recreation^56,57^, habitat and migration of specific species^58^, scenic beauty^59^, and economic development^60–62^. For the ecological network's compound function, it is necessary to build a multi-functional evaluation and coupling mechanism to solve functional coupling better^61–63^, while also managing conflicts between protection and construction^64,65^ and improving the rationality of ecological network identification and protection^66,67^.

### Exploration of interdisciplinary methods

The data quantification method was used to identify ecological networks mostly used in ecological planning^39,41,47,68,69^. However, the cross-disciplinary nature of GSSP is in line with a broader development trend in China^31,70–72^ and elsewhere^73–79^. In China, the focus of GSSP has also expanded from central urban areas to larger-scale areas^8^. Since 2019, China has begun to compile provincial TSP, and provincial-level GSSP has become an important research area^17^. The multi-functional requirements need to explore more interdisciplinary planning methods.

### Expansion of application areas

Besides being applied in GSSP, the ecological network and node identification can be used in many related planning areas. For example, the ecological red lines plan, the green infrastructure planning, the location of environmental restoration areas, and the projects that have an essential impact on the ecology, etc. The field of application needs to be further expanded.

## Conclusion

Research results showed that the quantitative, multi-method process succeeded in effectively identifying green corridors, blue corridors, ecological networks, and ecological nodes. Moreover, the regional situation of the identified networks and nodes were consistent with their characteristics. Especially the blue network identification method is of value, as blue corridors could be identified through DEM data in areas lacking hydrological data.

However, there are still some restrictions on the construction of provincial ecological networks. When striving for green infrastructure or system planning grounded in sound, some subjective factors may accompany the presented approach. It may happen in, for example, the assignment of resistance values, the setting of scenario simulation, the identification of ecological nodes, etc. These steps need to make artificial judgments with a good understanding of the current situation. Furthermore, as a provincial plan, it is mainly used as a guiding plan which can't be very accurate, and there is a certain degree of flexibility. For example, this article did not specify the size of the ecological network and nodes to reserve adjustment space for further constructive planning. More work is needed in order to find a better balance between objective data, subjective judgments and consideration of values, and regional specificity to improve the planning process. Besides, how to more scientifically couple the ecological network's functions and guide the actual construction work needs further research.
